# Cardiovascular Effects of Excess Growth Hormone: How Real is the Threat?

**DOI:** 10.31083/j.rcm2404095

**Published:** 2023-03-23

**Authors:** Frederick Berro Rivera, Marianne Katharina Taliño, Marie Francesca Ansay, Gerard Francis Mangubat, Mer Lorraine Mahilum, Rajiv Hans Menghrajani, Siena Placino, Sung Whoy Cha, John Paul Aparece, Marc Gregory Yu, Michael Lawrenz Co, Edgar Lerma, Krishnaswami Vijayaraghavan, Peter A. McCullough

**Affiliations:** ^1^Department of Medicine, Lincoln Medical and Mental Health Center, Bronx, NY 10451, USA; ^2^Ateneo de Manila School of Medicine and Public Health, 1604 Pasig City, Philippines; ^3^Department of Medicine, Southern Philippines Medical Center, 8000 Davao City, Philippines; ^4^St. Luke’s Medical Center College of Medicine - William H. Quasha Memorial, 1102 Manila, Philippines; ^5^Cebu Institute of Medicine, 6000 Cebu City, Philippines; ^6^Section of Vascular Cell Biology, Joslin Diabetes Center and Harvard Medical School, Boston, MA 02215, USA; ^7^Section of Cardiology, Thomas Jefferson University, Philadelphia, PA 19107, USA; ^8^Section of Nephrology, University of Illinois at Chicago College of Medicine/Advocate Christ Medical Center, Oak Lawn, IL 60612, USA; ^9^University of Arizona, Phoenix, AZ 85004, USA; ^10^Truth for Health Foundation, Tucson, AZ 85728, USA

**Keywords:** growth hormone, insulin-like growth factor-1, cardiovascular diseases, acromegaly

## Abstract

Patients with acromegaly carry a high risk of developing cardiovascular diseases (CVD). In fact, CVD is the leading cause of mortality among this group of patients. The most frequent cardiovascular complications are heart failure (HF), valvular disease, hypertension, arrhythmias, and coronary artery disease (CAD). The pathophysiology centers on the family of growth hormone (GH). These hormones are involved in normal cardiac development and function; however, excess of insulin-like growth factor-1 (IGF-1), the principally active hormone, can also cause negative effects on the cardiovascular system. HF in acromegaly usually presents with biventricular enlargement and diastolic dysfunction and is strongly associated with the duration of GH excess rather than the degree of hormone elevation. There is a high prevalence of valvular disease affecting aortic and mitral valves among patients with longer disease duration. The development of hypertension in acromegaly may be attributed to the effects of chronic GH/IGF-1 excess on different organ systems, which act via several mechanisms. The aspect of arrhythmia and CAD complicating acromegaly are currently not fully understood.

## 1. Introduction

**Fig. 1.**
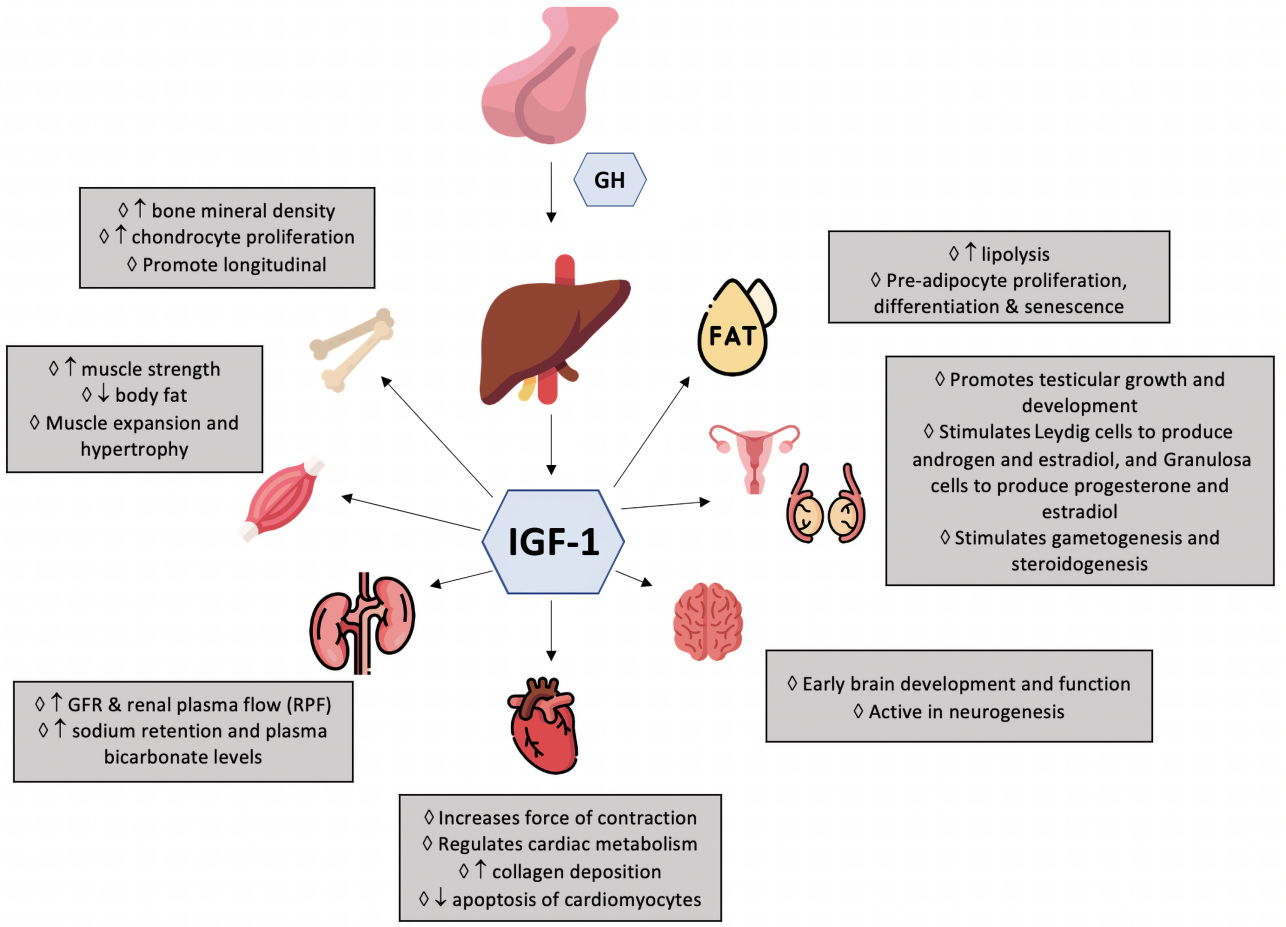
**Effects of GH/IGF-1 on different organs in the body**.

Growth hormone-releasing hormone (GHRH) from the hypothalamus signals the anterior pituitary to release growth hormone (GH) which in turn binds to its receptor in the liver and initiates the expression of insulin-like growth factor-1 (IGF-1). IGF-1 exerts its function through endocrine, paracrine, and autocrine signaling pathways [1, 2], and is responsible for DNA, RNA, and protein synthesis in the bone and muscle, cell growth, differentiation, and proliferation, and myriad other bodily functions (Fig. 1) [3]. An excess in circulating GH and IGF-1, most commonly due to a pituitary adenoma and rarely due to ectopic GH secretion or GHRH excess, leads to acromegaly [4]. Acromegaly is a chronic, multisystem disease with a wide range of manifestations, which may be due to a direct effect of the lesion or a long-term effect of excess GH and IGF-1 on organs and tissues [5]. Early manifestations may be subtle, and progression may be slow that patients and caregivers may not immediately recognize these changes as pathologic [6]. These factors contribute to the significant delay in diagnosing acromegaly, with a mean diagnostic delay of 5.5 years [7]. According to the ACRO-POLIS study by Caron *et al*. [6] in 2019, the most frequently reported symptoms 6–10 years prior to diagnosis are enlarged hands and feet, snoring, menstrual cycle changes, weight gain, and carpal and/or cubital tunnel syndrome. The diagnosis of acromegaly is typically confirmed with measurement of IGF-1 levels in patients with typical manifestations such as acral and facial features. However, recent guidelines recommend IGF-1 level measurement to also include those without typical manifestations but with several atypical signs such as sleep apnea, type 2 diabetes mellitus, debilitating arthritis, carpal tunnel syndrome, and hypertension [8]. Serum IGF-1 levels are preferred over GH levels as biomarker the in the diagnosis of acromegaly due to several factors: significantly longer half-life of approximately 15 hours compared to less than an hour for GH, the episodic nature of GH secretion, and a closer correlation of IGF-1 levels with the onset of clinical signs, wherein an elevated IGF-1 with normal GH reflects an earlier stage of the disease [9]. IGF-1 levels may be falsely increased in pregnancy and late adolescence, which reinforces the need for a standardized age and sex-matched IGF-1 levels specific for each of the assays used in measurement. On the other hand, falsely decreased levels may be seen in hepatic or renal failure, hypothyroidism, malnutrition, severe infections, poorly controlled diabetes, and in patients using combined oral contraceptive pills. If factors causing false variations of IGF-1 levels are present or if IGF-1 assays are not available, a GH suppression test with a 75 g oral glucose load can be performed as a confirmatory test [8].

Pituitary Magnetic Resonance Imaging (MRI) is recommended for all patients with acromegaly to determine the presence of any macro- or microadenomas [10]. In patients with biochemically confirmed acromegaly with a normal pituitary MRI, further testing with GHRH measurement, somatostatin receptor scintigraphy, and thoracic or abdominal imaging may be considered [8].

Cardiovascular diseases (CVD) is the leading cause of mortality in patients with acromegaly [10, 11, 12]. The most frequent cardiovascular complications in acromegaly are heart failure (HF), valvular disease, hypertension, arrhythmia and coronary artery disease (CAD) [13]. Risk factors for these cardiac abnormalities include longer duration of GH hypersecretion, increasing age and higher Body Mass Index (BMI) [13]. On top of the direct cardiovascular risk from prolonged GH hypersecretion, patients with acromegaly are further predisposed to develop other significant cardiovascular risk factors such as diabetes, dyslipidemia, obesity and sleep apnea [13]. The presence of any cardiovascular comorbidities at the time of acromegaly diagnosis is associated with significantly increased odds of hospitalization and risk of death [13]. Prolonged delay in diagnosis is also associated with significantly increased morbidity and mortality due to cardiovascular complications [7].

## 2. Epidemiology and Clinical Presentation of Acromegaly

Globally, the prevalence rate of acromegaly is about 59 cases per 1,000,000 people, while the incidence is 3.8 cases per 1,000,000 people [13]. While different studies have shown some variability in sex distribution, the prevalence seems to be equal among men and women on average. Similarly, the prevalence in different countries may be affected by population size and geographical area. For instance, Lavrantaki *et al*. [14] based their study from the published data done in Europe and found the annual incidence to be between 2 to 11 cases per 1,000,000 people, while the prevalence was between 28 to 137 cases per 1,000,000 people. In Korea, on the other hand, the average 5-year incidence (2013–2017) was 4.2 cases per million, and the prevalence was 32 cases per million per year [15].

Patients are diagnosed at the fourth to fifth decade of life on average; however, there is a delay of 5 to 8 years before diagnosis is established [14, 16]. The most common manifestations of acromegaly are acral enlargement and coarse facial quality with 78.8–85.7% and 71.2–71.4% respectively [11]. Other signs and symptoms include macroglossia (46.2%), sweating (44.2–51.7%), skin thickening (38.5%), snoring (28.6%), arthralgias (28.6–50%), tiredness (14.3–38.5%), carpal tunnel syndrome (14.3%), and visual disturbances (34.6%) [14].

Patients may present with acute and/or chronic complications from acromegaly [17]. A study in New Zealand listed sweating, carpal tunnel, headache, visual field defect and sleep apnea as acute complications, while hypertension, diabetes, CVD and arthropathy were listed as chronic complications. The Liège Acromegaly Survey (LAS) Database in 2017 found that 28.8% of patients have hypertension; 27.5% with diabetes mellitus; 25.5% with sleep apnea; 15.5% with cardiac enlargement; and <5% with stroke, arrhythmias, ischemic heart disease, HF, and myocardial infarction. Other comorbidities that may be present in patients include thyroid nodule or goiter (34%), colonic polyps (13%), osteoporosis (12.3%), and cancer (breast, thyroid, and skin) (1%) [18]. The study showed that the prevalence of these complications decreased by 5–40% for acute complications and 10–20% for chronic complications after treatment when the final GH was <2 ug/L [16, 17]. Renehan & Brennan [19] emphasized the association of acromegaly with colon, rectal and thyroid cancers, with respective risk ratios of 2.46 (95% CI 1.79, 3.38), 1.41 (95% CI 0.54, 3.71), and 3.64 (95% CI 1.63, 8.11).

Acromegaly may be also associated with certain conditions including multiple endocrine neoplasia (MEN)-1, extrapituitary tumors (pancreas, lung, ovary), McCune-Albright Syndrome, Carney Syndrome, isolated familial somatotropinomas, and familial isolated pituitary adenoma (FIPA) [16]. Most patients have macroadenomas (66.7–88.9%) at the time of diagnosis, which could be likely due to the delay in diagnosis. Moreover, few cases (14.3–31.8%) were due to microadenomas. The different presenting sizes of adenomas may pose as a challenge for surgical interventions [14]. A significant increase in epicardial fat thickness (9.71 ± 1.54) in remission acromegaly (RA) and (10.08 ± 1.95 mm) in active acromegaly (AA) vs. controls (5.74 ± 0.92 mm, *p *< 0001), significantly decreased aortic strain and aortic distensibility (3.81 ± 1.94) in RA and in AA (3.68 ± 1.99) vs. controls (8.19 ± 4.19%, *p *< 0.0001), and increased aortic root diameter at the sino-tubular junction (30 ± 4 vs. 26 ± 3 mm, *p* = 0.0001) and the ascending aorta (33 ± 5 vs. 30 ± 4 mm, *p* = 0.006) warrant a more extensive cardiovascular evaluation (echocardiography, cardiac MRI) in the presence of these features [20, 19]. In a cohort study by Wu *et al*. [21] in 2020, 1195 patients displayed excessive risk of mortality (41% more) over a 17-year period, with higher risk in early-onset disease. Excessive circulating GH and IGF-1 in acromegalic patients induce comorbidities such as CVD (atherosclerosis, cardiomyopathy, arrythmias), diabetes, and cancers (digestive, respiratory, breast and lymphoma), which are direct contributors of higher mortality in acromegaly patients [21]. If left untreated, mortality occurs in almost 100% of patients within 15 years from CVD, and only 20% of patients with diabetes and acromegaly will survive 20 years [22]. For treated patients who survived more than 5 years in early and middle age onset groups, mortality rates were similar with the general population, emphasizing the importance of aggressive and early treatment which potentially neutralizes the associated mortality risk, especially with the advent of pituitary surgery and radiotherapy [21].

## 3. Overview of Cardiac Effects of GH and IGF-1

**Fig. 2.**
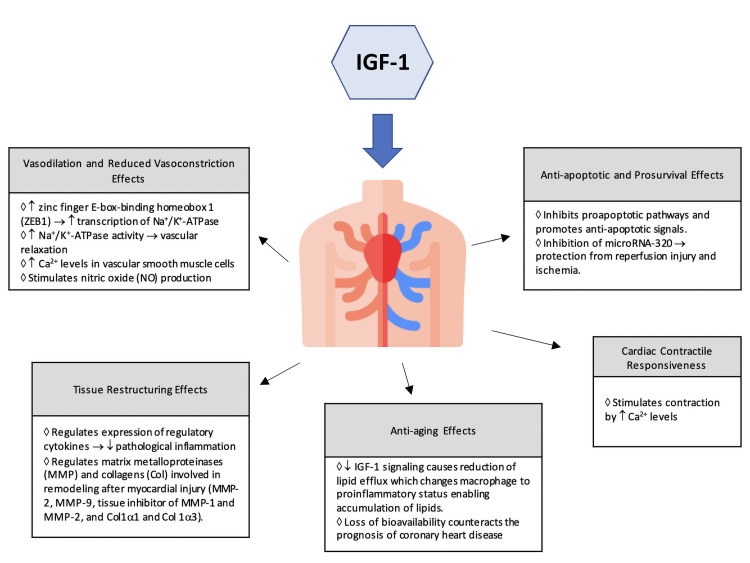
**Effects of IGF-1 on the Cardiovascular System as seen *in vivo* studies**.

**Fig. 3.**
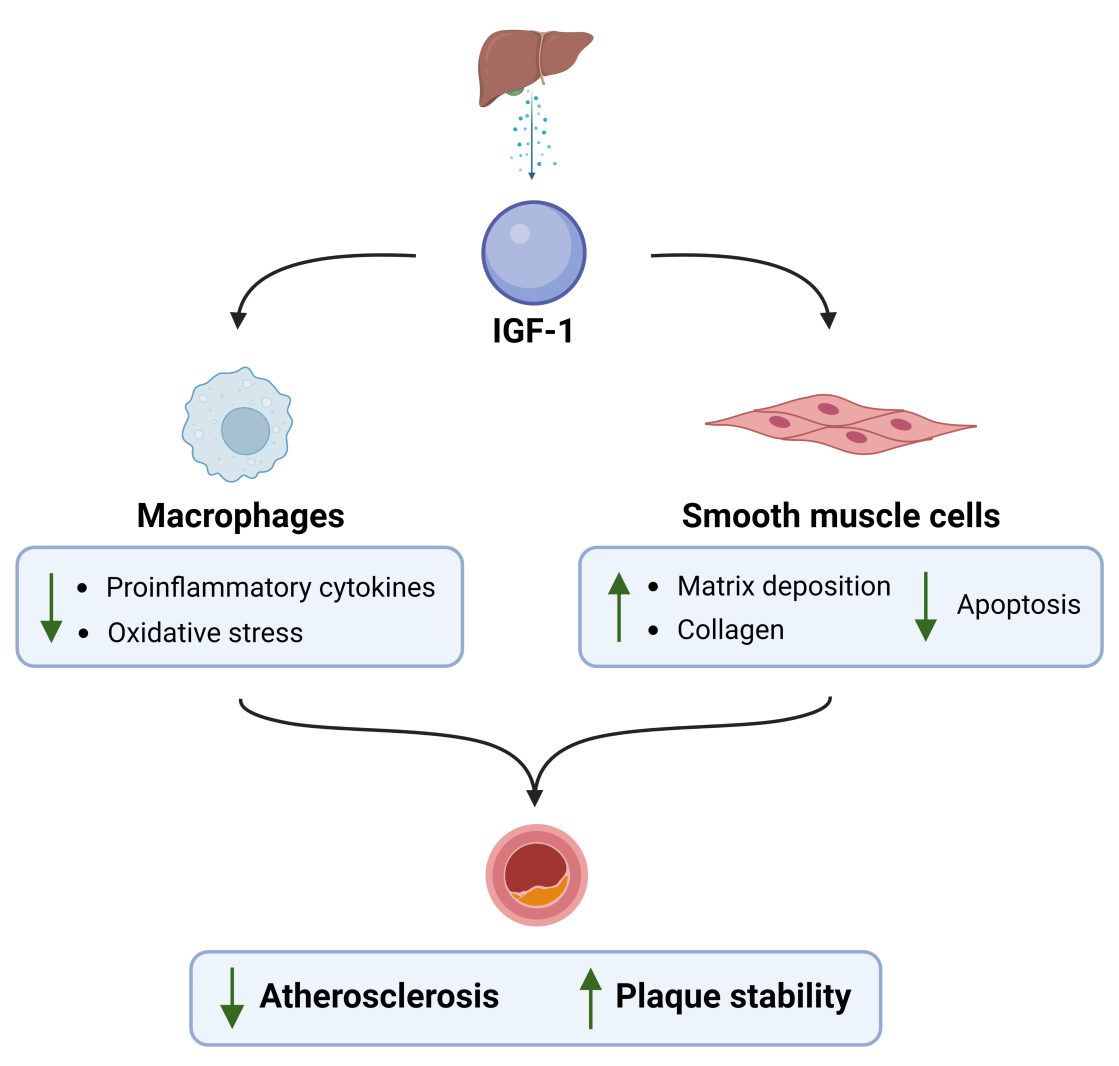
**IGF-1 effects on macrophage and smooth muscle cell decreasing atherosclerosis. Adapted from Higashi *et al***. **[** 31 **]** .

The effects of GH and IGF-I on the cardiovascular system have been studied in animal models and human myocardial tissue. The GH and IGF-I receptors are expressed at high levels in both myocardial tissue and blood vessels [23]. Animal studies have demonstrated that IGF-1 had a physiologic, antiapoptotic, and pro-survival effects on the heart and is associated with improved cardiac function, alleviation of high-fat diet-induced cardiac dysfunction, regeneration of the myocardium following infarction, and attenuation of endothelin receptor A (ETA)-mediated coronary contraction [2, 24, 25, 26, 27, 28]. Short term exposure of the normal heart to high GH and IGF-I concentrations can enhance myocardial contractility and relaxation [29]. Obradovic *et al*. [2] discussed the effects of IGF-1 on the cardiovascular system in a review article summarized in Fig. 2. Low IGF-1 or GH levels were associated with a twofold increased risk for ischemic heart disease due to its correlation with obesity and sedentary lifestyle [30]. IGF-1 has been shown to have atheroprotective effects in a study by Higashi *et al*. [31], where an inverse correlation of systemic IGF-1 levels and atherosclerosis was demonstrated by infusing IGF-1 in ApoE-null mice. The presence of IGF-1 was shown to induce phenotypic changes in plaques, represented by attenuated cytokine expression, decreased macrophage counts, decreased oxidative stress, and increased presence of collagen and smooth muscle cells (Fig. 3, Ref. [31]). In human myocardial tissue, activation of IGF-1 receptors demonstrated a concentration-dependent positive inotropic effect secondary to increased intracellular ${\mathrm{Ca}}^{2+}$, enhancement of L-type ${\mathrm{Ca}}^{2+}$ currents and ${\mathrm{Na}}^{+}$–$H^{+}$ exchange. GH, in contrast, does not influence ${\mathrm{Ca}}^{2+}$ currents in acute settings; however, it has been shown to increase peak intracellular ${\mathrm{Ca}}^{2+}$ concentrations after long term exposure *in vitro* [32].

Excess IGF-1, however, can also have negative effects on the cardiovascular system. Increasing the presence of circulatory smooth muscle cells, as seen in animal models, can lead to restenosis, pulmonary hypertension, and vein graft failure [31]. Atherosclerosis in acromegaly is controversial since there is not much evidence on the effect of IGF-1 on forming atheromas [33]. In the work of Ito *et al*. [34], IGF-I was found to possibly induce hypertrophy in cultured neonatal rat cardiomyocytes. In a study done in 2017 by Cansu *et al*. [35], incidences of atherosclerosis, left ventricular hypertrophy (LVH), and diastolic dysfunction were more common in patients with acromegaly, both controlled and uncontrolled.

Pathologic abnormalities seen in an acromegalic heart include ventricular enlargement, interstitial fibrosis, cardiomyocyte degeneration, myofibrillar derangements, leukocyte infiltration and increased collagen deposition [22, 33]. Colao’s review article mentioned that aging and prolonged duration of excess GH/IGF-1 levels are determinants of cardiac derangement [36]. Cardiac hypertrophy was seen in patients who had acromegaly for an extended duration and was more common in patients aged >50 years. Because of this, it was suggested that cardiac hypertrophy may be an early manifestation of acromegaly, with a direct correlation between the severity of cardiac hypertrophy and duration of acromegaly [36]. These structural changes increase the risk of both right and left ventricular (LV) dysfunction leading to HF. Rhythm disturbances including atrial fibrillation are also more common in acromegaly patients compared to the general population, as IGF-1 can directly affect myocardial contractility by increasing intracellular ${\mathrm{Ca}}^{2+}$ levels [36]. Excessive GH/IGF-1 also contributes to diastolic and systolic dysfunction, which could lead to HF [37].

The sections below will individually discuss the different cardiovascular abnormalities which may be present in acromegaly patients.

## 4. Cardiovascular Diseases Associated with Acromegaly

### 4.1 Myocardial Mechanics in Acromegaly

GH and IGF-1 cause changes in cardiac morphology and function either directly by affecting myocyte growth and contractility, or indirectly through other mechanisms [12, 38]. In acromegaly patients, myocardial hypertrophy occurs even in the absence of hypertension and in young patients, emphasizing the direct impact of GH and IGF-1 in myocardial mechanics. Indirectly, comorbidities further aggravate cardiac hypertrophy. One proposed mechanism is insulin resistance, where induction of myocardial hypertrophy is related to the structural similarity of insulin to IGF-1 and its ability to stimulate IGF-1 receptors [39].

The most common cardiac change in acromegaly is biventricular hypertrophy, with majority of patients showing obvious LVH at the time of diagnosis. These changes eventually lead to diastolic dysfunction, and more rarely, systolic dysfunction [22]. The incidence of diastolic dysfunction is reported in an average of 46.3%, while progression to systolic dysfunction is observed in less than 3% of patients [12, 40].

In a study performed in Poland, acromegaly patients presented with a higher LV mass (132 vs. 108 g/m, *p *< 0.001), lower mitral annulus velocity (septal value: 8 vs. 9.9 cm/s; *p* = 0.004), lower left ventricular ejection fraction (LVEF) (63.4 vs. 66.9%, *p *< 0.001) and lower global longitudinal strain (GLS) (mean: –18.1 vs. –19.4%, *p* = 0.023) than control. Left atrial morphology was also found to be impaired, with greater AP diameter (40.3 vs. 36.9 mm, *p* = 0.003) and left atrial volume index (37.9 vs. 27.6 mL/$m^{2}$, *p *< 0.001) in acromegaly patients [38]. More recently, another study done in Brazil showed acromegalic patients to have higher prevalence of LVH (40% vs. 19%, *p *< 0.01) and higher LV mass (87.9 g/m ± 27 vs. 69.3 g/m ± 17.5, *p* = 0.001) than control, but LVEF (65.16% ± 5.99 vs. 62.9% ± 7.41, *p* = 0.19) and GLS (–16.74 ± 3.18 vs. –16.6 ± 3.42, *p* = 0.909) were found to be similar across both groups [41].

Right atrial morphology and function is also affected in acromegaly. In a three-dimensional speckle-tracking echocardiography (3DSTE) analysis, all volumetric parameters were significantly higher in acromegaly patients (Vmax = 54.5 ± 14.4 vs. 47.2 ± 13.5; Vmin = 35.5 ± 10.2 vs. 28.7 ± 9; VpreA = 45.1 ± 11.1 vs. 37.9 ± 11) compared to control [42].

In summary, GH and IGF-1 directly affect myocardial mechanics in acromegaly, with concentric hypertrophy of both ventricles, more prominently the left, resulting in diastolic dysfunction and rarely systolic dysfunction. Recognition of even the smallest abnormalities of myocardial mechanics can lead to earlier effective risk mitigation strategies.

### 4.2 Heart Failure in Acromegaly

The prevalence of acromegalic cardiomyopathy is estimated to be around 3% [10]. Prevalence of HF in acromegalic patients was found to be significantly increased (8.6%) in one study involving Korean subjects, with a mortality of 14.5% [43]. Hong *et al*. [44] similarly concluded that patients with acromegaly had a higher incidence of atrial fibrillation (3.06 vs. 1.70; *p* = 0.001), congestive HF (3.11 vs. 1.63; *p *< 0.001) and all-cause mortality (6.31 vs. 4.03; *p *< 0.001). It is a unique form of HF seen almost exclusively among patients with GH excess, characterized by enlargement of both ventricles leading to LV diastolic dysfunction, septal hypertrophy, myocardial inflammation, necrosis and fibrosis [10, 12, 45]. Interestingly, this phenotype of HF is more affected by the duration of GH excess than the degree of elevation [10]. In health, short term elevation in GH and IGF-1 upregulates muscle cell contractile mechanism by increasing calcium influx thus increasing cardiac muscle contractility [10]. The pathogenesis involves the direct effect of GH and IGF-1 excess on the heart muscle as well as indirect mechanisms such as arterial hypertension and abnormalities in glucose and lipid metabolism [12]. GH elevation brings about positive inotropy in the early stage and negative inotropy and myocardial dysfunction in prolonged or later stage [12]. Histopathologic examinations on heart specimens showed increased collagen deposition, derangement of myofibrils and mononuclear infiltration [10, 45]. Acromegalic cardiomyopathy is characterized into three stages (See Table 1) [10, 12, 45]. The first stage is characterized by hyperkinetic LV leading to increased contractility and increased cardiac output and heart rate [12, 45]; the abnormalities seen at this stage are still reversible [12]. The second stage is evidenced by diastolic dysfunction and appearance of systolic dysfunction on effort [12]. Cardiac biopsy in this stage would reveal fibrosis and progressive hypertrophy [45]. The third stage or the end stage is marked by congestive HF from both systolic and diastolic dysfunction [45]. Patients are usually diagnosed during the second stage [13]. Newer studies using cardiac MRI show a lesser prevalence of acromegalic cardiomyopathy compared to the older studies which utilized 2D echocardiography [10]. Early treatment of hormone excess either by pituitary surgery or medical therapy showed regression of the histopathologic changes and improvement of LV dimension [10, 12]. Progression from early disease to frank congestive HF is uncommon; however, the global prognosis among patients in stage 3 disease is usually poor [12, 45]. Overall, the 1- and 5-year mortality rates for patients on stage 3 were 25% and 37.5%, respectively [12].

**Table 1.** **Stages of acromegalic cardiomyopathy and corresponding clinical and hemodynamic features**.

Stage	Clinical features	Hemodynamic features	Years of active disease
First stage (reversible stage) (“hyperkinetic stage”)	∙ Increased inotropy	∙ Increased cardiac output	<5 years
	∙ Increased heart rate	∙ LV hyperkinesia (“hyperkinetic syndrome”)	
	∙ Decreased peripheral vascular resistance		
Second stage	∙ Progressive hypertrophy (cardiomegaly)	∙ Diastolic dysfunction	>5 years
	∙ Most patients are diagnosed to have heart failure	∙ Systolic dysfunction on effort	
	∙ Exercise intolerance		
Third stage	∙ Clinical congestion is usually present	∙ Systolic and diastolic dysfunction	>15 years
		∙ Valvular abnormalities (mitral and aortic regurgitation)	
		∙ Cavity dilation	
		∙ Systolic and diastolic failure	
		∙ Right ventricular (RV) involvement	

### 4.3 Coronary Artery Disease in Acromegaly

Currently, data on the risk of CAD among acromegaly patients are conflicting. In the classic study of Colao *et al*. [46], they measured the intimal-media thickness (IMT) using M-mode ultrasonography among patients with active disease, cured from acromegaly and controls. They found out that IMT was significantly higher among patients with active disease and those cured compared to controls [46].

However, the prevalence of true carotid plaques was not increased in patients with acromegaly compared to controls, suggesting that vessels are not significantly affected by prolonged excess of GH and IGF-1 [46]. In another study by Paisley *et al*. [47], patients with acromegaly and 46 healthy controls underwent evaluation of aortic pulse wave velocity (PWV) and IMT. They found out that patients with acromegaly had independently increased aortic PWV but had normal or unchanged carotid IMT compared with controls [47]. They concluded that premature CVD in patients with acromegaly are more likely pressure-related rather than a true atherosclerotic heart disease.

Patients with acromegaly usually present with significant additional risk factors for coronary heart disease, including hypertension and diabetes mellitus [32]. In a study done by Berg *et al*. [48], prevalence of hypertension and diabetes was significantly higher in patients with active acromegaly compared with age- and gender-matched controls. In addition, acromegalic patients had lower HDL and LDL cholesterol levels, whereas triglyceride levels were not different from the normal population. Thus, control of these comorbid risk factors as well as the GH and IGF-1 levels are essential to reduce the likelihood of developing CAD [45, 48].

### 4.4 Valvular Heart Disease in Acromegaly

Acromegaly is associated with an increased prevalence of valvular heart disease [49]. This increase is directly proportional to the degree and duration of GH and IGF-1 elevation [49]. In the classic study by Pereira *et al*. [49], using 2D echo with doppler studies, they found out that significant valve disease was prevalent among patients with acromegaly compared to controls (22% vs. 6.7%, *p* = 0.005). In that same study, aortic and mitral valve regurgitation was significantly more prevalent [50, 39]. There was an increase in odds of 19% for the development of valvular disease for every additional year of exposure to tonically elevated GH concentrations. The study of Natchev *et al*. [39]. found aortic regurgitation in 31%, mitral regurgitation in 47%, and tricuspid regurgitation in 37% of their cases.

In another study by Colao *et al*. [50], M-mode, 2D and pulsed doppler echocardiography was performed to characterize the aortic and mitral valves. The authors determined that the prevalence of valve abnormalities was significantly higher in both patients with active disease and cured for at least year compared to controls [50]. The pathogenesis of this myxomatous heart valve disease remain uncertain, but current theories suggest that it is due to activation of the interstitial tissue with subsequent collagen degradation, fragmentation of elastin and glycosaminoglycan accumulation, leading to leaflet thickening and redundancy [49].

### 4.5 Hypertension in Acromegaly

Hypertension is a well-known comorbidity in patients with acromegaly. In a retrospective study conducted in Pakistan from 2000–2020 by Khan *et al*. [51], out of 89 patients enrolled in the study, 32.95% were identified to be hypertensive, defined as a systolic blood pressure (SBP) of ≥140 mm Hg or diastolic blood pressure (DBP) of ≥90 mm Hg. Additionally, 3.37% were classified as pre-hypertensive with a SBP of 120–139 mm Hg and DBP of 80–89 mm Hg [51]. These findings were comparable to the prevalence reported by a study by AV Dreval *et al*. [52] and Espinosa-de-los-Monteros *et al*. [53] with 50% and 31.9% in Russian and Mexican populations respectively.

The development of hypertension in acromegaly may be attributed to the effects of chronic GH/IGF-1 excess on different organ systems, which act via three main mechanisms described by Puglisi *et al*. [54]. First, the expansion of extracellular fluid volume secondary to sodium and water retention by the kidney. Second, the increase of peripheral vascular resistance, which may explain the preferential increase in DBP as opposed to SBP amongst acromegalic patients. It has been demonstrated in several studies that there is an inverse correlation between the levels of GH/IGF-1 and nitric oxide (NO), which is responsible for the vasodilatory properties of the endothelium [55]. Furthermore, the presence of both IGF-1 and insulin may give rise to the development of vascular hypertrophy through the activation of the Renin-Angiotensin-Aldosterone-System (RAAS). This may explain the hypertrophic remodeling of subcutaneous small resistance arteries of acromegalic patients compared to the eutrophic remodeling in essential hypertension [56]. Lastly, the development of sleep apnea syndrome which occurs in 45–80% of acromegalic patients may lead to loss of physiologic nocturnal blood pressure dipping in patients. The cyclic period of desaturation and reoxygenation in sleep apnea causes vasoconstriction of vessels, which in turn exacerbates hypertension [54].

As shown in studies, while hypertension in patients with acromegaly is usually not severe, it still plays a key role in the development of other comorbidities such as diabetes, insulin resistance, and other CVD [57]. Bielohuby *et al*. [58] associated aldosterone elevation to chronic GH excess rather than IGF-1 in transgenic mouse models, leading to hypertension as well as potentially increased CVD risk in patients with acromegaly. Acromegalic patients also present with enhanced epithelial sodium channel (ENaC) activity, which contributes to secondary soft tissue swelling and increased extracellular volume, leading to hypertension. Hypertension can also increase mortality by 3.3-fold in patients with acromegaly. In both logistic regression and Kaplan-Meier analyses of the same study, it shows that the presence of CVD impairs the survival of patients with both acromegaly and hypertension. This comorbidity is the most robust independent predictor of mortality in patients with acromegaly [57].

### 4.6 Arrhythmia in Acromegaly

Mortality in acromegaly may be attributed to arrhythmias and sudden cardiac death [59], although not much is known as of writing since these aspects of CVD are less studied in acromegaly [60]. According to a retrospective study by Khan *et al*. [51] on the prevalence of comorbidities in patients with acromegaly, while many studies reported arrhythmias, none of the patients in their own study had a rhythm disorder [61]. In a case series by Dutta *et al*. [61] on acromegaly with overt congestive HF, 50% of the patients died from ventricular arrhythmias, attributed to abnormal ventricular remodeling. Another case study by Subramnaian *et al*. [62] illustrated that idiopathic premature ventricular contraction (PVC) and ventricular tachycardia (VT) can be manifestations of any systemic disease, including acromegaly. Other rhythm disorders seen during physical exercise are paroxysmal atrial fibrillations, paroxysmal supraventricular tachycardia, sick sinus syndrome, bundle branch block, and atrial and ventricular ectopic beats [63].

Although few data are available on the prevalence and severity of arrhythmias in acromegaly patients, some studies attempted to explain the pathogenesis of these rhythm disorders in this population. As previously mentioned, IGF-1 has a direct positive inotropic effect on cardiac myocytes through increasing ${\mathrm{Ca}}^{2+}$ availability to the myofilaments [64]. The myocyte shortening (% of diastolic cell length) is dose-dependent with IGF-1, peaking at 150–300 ng/mL [64]. Furthermore, structural changes like LVH and fibrosis in acromegaly puts the patient at risk for arrhythmias, as collagen deposition in the cardiac tissue is also associated with cardiac rhythm disorders [65]. The slow and heterogeneous transmission of action potential in the heart is most likely due to the myofibrillar derangement and uncoupling of cardiomyocytes [65]. Another explanation for pathogenesis of arrhythmia in acromegaly is QT variability. A study by Orosz *et al*. [63] demonstrated that acromegaly patients have elevated beat-to-beat short-term QT interval variability, which can be used as a predictive measure or indicator of impending arrhythmia and/or sudden cardiac death. Electrocardiography (ECG) parameters were compared and measured in acromegaly patients and age-matched controls. The short-term variability of the duration of repolarization (STVQT) was shown to be significantly increased (4.23 ± 1.03 ms vs. 3.02 ± 0.80, *p *< 0.0001) in acromegaly patients than in the general population.

As aforementioned, the pathogenesis of arrhythmias in acromegaly is multifactorial. Thus, addressing these factors are the key in managing arrhythmias in patients with acromegaly.

## 5. Diagnosis of Cardiovascular Comorbidities in Acromegaly

### 5.1 Overview of Physical Examination Findings and Diagnostic Modalities

Given the subtleness of cardiovascular manifestations in patients with acromegaly, the importance of focused history and through physical examination cannot be overemphasized. Cardiac examination may reveal S3 and S4, crackles and LV heave [10]. In the setting of regurgitant valvular lesion, one can appreciate murmurs of aortic and mitral regurgitations. Echocardiography is the most important non-invasive tool for confirming LV dysfunction and valvular abnormalities [10]. Electrocardiogram and 24-h ECG Holter monitoring are also valuable in determining late potentials, premature ventricular complexes and other arrhythmias [60]. Finally, a comprehensive laboratory evaluation is warranted to rule out concomitant conditions such as hypothyroidism, renal failure, and anemia; and to assess for cardiomyopathic changes (hs-Troponin, brain natriuretic peptide, ST2, galectin-3). Table 2 shows the appropriate diagnostic tools for each cardiovascular comorbidity in patients with acromegaly.

**Table 2.** **Summary of the diagnostic tests that may be used to identify the different cardiovascular alterations in patients with acromegaly**.

Cardiovascular comorbidities	Diagnostic tool
Hypertension	At baseline and every 6 months or upon change of antihypertensive treatment
	ECG, annually if abnormal
	2D echo, annually if abnormal
Heart Failure	At baseline and every 6 months or upon change of antihypertensive treatment
CAD	
Valvular heart disease	

### 5.2 New Findings on Speckle Tracking Echocardiography and Cardiac MRI

Speckle tracking echocardiography (STE) using 2D and 3D echocardiography, and cardiac MRI are among the latest diagnostic tools widely available [66]. Assessment of myocardial deformation on strain echocardiography is also very valuable in the assessment of global and regional ventricular dysfunction [67]. The first study that explored the role of strain was the group of Koca *et al*. [67]. According to the study, LV and LA systolic functions in acromegaly patients were found to be abnormal on strain echocardiography despite normal LV systolic function on conventional echocardiography [67]. Furthermore, they found out that almost 50% of patients with acromegaly had silent LV dysfunction detected with strain echocardiography [67]. These findings were in contrast to the study of Oliveira *et al*. [68], which reported no difference in GLS among patients with acromegaly and control. More recently, the use of 3DSTE has been identified to overcome some of the limitations of 2DSTE, offering additional deformation parameters such as area strain and a detailed analysis of LV geometry and function from a single 3D acquisition. It is a validated method for LA quantification in patients with acromegaly as compared to 2DSTE and volumetric real-time 3D echocardiography. Although this method currently has a low feasibility for everyday practice, there is a growing interest for this technique and it has been recognized to be the potential gold-standard tool assessing LV systolic function in the near future [69, 70]. On the other hand, the use of cardiac MRI in the cardiac evaluation among patients with acromegaly is limited. Guo *et al*. [71] used cardiac MRI to determine the frequency and severity of cardiac structural and functional abnormalities. Myocardial fibrosis was detected in 15% of patients, mainly in the middle layer of the myocardium [71]. The authors concluded that cardiac MRI is more accurate for acromegaly patients with stage 3 disease than echocardiography. Table 3 shows the comparison between STE and cardiac MRI.

**Table 3.** **Advantages and disadvantages of different cardiac imaging modalities in the diagnosis of cardiac involvement of acromegaly**.

Cardiac imaging modality	Advantages	Disadvantages
GLS/2DSTE	∙ Available everywhere	∙ Needs good image
	∙ Low-cost	∙ Does not detect myocardial fibrosis
3DSTE	∙ Offers additional parameters	∙ Low feasibility in everyday practice
	∙ Eliminates directional limits of 2D speckle tracking	∙ Accuracy highly depends on technical skills of operator
	∙ Time-saving	∙ Not yet readily available in developing countries
Cardiac MRI	∙ Higher spatial resolution	∙ Expensive
	∙ Gold standard for the assessment of myocardial mass	∙ Not readily available in developing countries
	∙ Can precisely detect myocardial fibrosis	∙ Cannot be used to patients with metal implanted on their bodies
	∙ Can precisely assess RV systolic function	

## 6. Treatment and Prognosis

Cardiovascular mortality in acromegalic patients depends on three main factors: GH levels, severity of arterial hypertension, and CVD. It has been shown that a reduction in serum GH concentrations to less than 1–2 μg/liter and normalization of serum IGF-I levels reduce mortality to a level similar to that of the general population [72]. Overall, normalization of GH/IGF levels either with pharmacotherapy or surgery improves cardiac mortality of acromegaly patients [72]. Well-controlled, middle-aged, acromegalic patients are more likely to have reversible cardiomyopathy than those with long-standing GH/IGF-1 elevation [50].

Studies suggest that the early initiation of somatostatin analogue (SSA) such as Octreotide LAR can potentially arrest or regress cardiac abnormalities and normalize GH/IGF-1 levels. In a case study, an acromegalic patient with chronic HF started on a daily dose of Octreotide showed an improved cardiac function including a decrease in LV wall thickness from 22.5 to 17.8 mm and an increase in systolic ejection fraction from 38 to 50% [73]. Its effect on arrhythmia has been particular on decreasing the frequency of premature ventricular complexes (PVC). In two separate case studies, Holter 24-hour electrocardiographic monitoring revealed improvement from 17,249 to 2882 beats a day and 24,277 to 2062 beats a day after Octreotide treatment. It has been hypothesized that Octreotide’s reduction of the hypertrophied cardiac tissue may have contributed to the improvement of PVCs [73, 74].

Pegvisomant (PEG) has also been shown to improve cardiac structure and function and reduce prevalence of arrhythmia and Framingham risk score. Combined therapy of SSA and PEG for 12 and 60 months showed significant improvement of cardiac structure and performance in terms of cardiac LV mass index and ventricular filling velocities, compared with long-term use of SSA alone. Its mechanism in acromegalic arrhythmias is still unclear; nonetheless, a study by Auriemma *et al*. [75] described that the effect may lie on the direct action of PEG on the pacemaker cells and on membrane calcium channels by working on the GH receptors in the conduction system. In this study, it was illustrated that PEG reduced the prevalence of arrhythmias by 50% (from 15 to 7.7%) and its complete disappearance was observed in one patient after an 18-month treatment. In conclusion, the study demonstrated that long-term treatment with PEG decreases a patient’s mean, minimum, and maximum heart rates and improves rhythm conduction abnormalities in acromegalic patients [75].

Cardiovascular effects of Cabergoline therapy on acromegalic patients remain to be unclear as it may be dose and duration dependent. In a study by Maione *et al*. [76], a median cumulative dose of 203 mg Cabergoline was used for a median of 35 months versus those who had never received Cabergoline. Results revealed a similar incidence of new valve regurgitation (40.0 and 45.8%, *p*-value: 0.68) and disease control [76].

Additionally, management of hypertension, dyslipidemia, diabetes, and other risk factors in acromegaly patients must be based on guidelines in the treatment of hypertension in the general population [66].

## 7. Current Problems and Possible Future in Acromegaly Management

The current first-line treatment for acromegaly is transsphenoidal surgery (TSS), but remission is only seen in approximately half of patients, even in reference centers. Other available options include medical and radiotherapy. However, despite the multiple options for treatment, approximately 40% of patients remain uncontrolled [77]; and more importantly, regardless of biochemical control, health-related quality of life remains unimproved [78]. There is often a delay in treatment owing to delayed diagnosis as well, as symptom development in acromegaly can take years [79]. The current treatment for acromegaly is based on a “trial and error” approach, as no clear biomarkers or other treatment predictors are readily available to guide therapy with high accuracy. As such, studies are looking into innovations in treatment to a precision medicine which can direct treatment in a more individualized approach. Moreover, new drugs for acromegaly are being developed, with potential to further improve disease control [78]. Lastly, attempts at earlier diagnosis and treatment can decrease progression of disease and its associated comorbidities.

## 8. Conclusions

Cardiovascular complications of acromegaly can present as HF, arrhythmia, valvular disease, and hypertension. The severity of these complications is almost always correlated to the severity and duration of GH and IGF-1 excess. A thorough history and focused physical examination are not enough to determine the extent of the cardiac complications; hence, newer diagnostic modalities such as cardiac MRI and GLS are increasingly being used. Guideline-directed medical therapy as well as appropriate control of excess hormone levels can control and even reverse the cardiovascular complications in this population. Opportunities exist in developing a new drug to effectively balance GH/IGF-1 levels, as this will be of great benefit in the management of HF subjects with acromegaly. Further study is warranted to also assess the benefits of this strategy in acromegaly patients with other organ involvement, such as those with nonalcoholic steatohepatitis/nonalcoholic fatty liver disease (NASH/NAFLD).
